# The Dubousset functional test: a reliable and validated physical function and balance assessment tool for older Chinese adults

**DOI:** 10.1186/s12877-025-06703-0

**Published:** 2025-11-21

**Authors:** Zhizhuo Wang, Cheng Lin, Jing Yang

**Affiliations:** Department of Rehabilitation Medicine, School of Health, Fujian Medical University, Fuzhou, 350122 China

**Keywords:** The Dubousset functional test, Physical function, Balance, Older adults

## Abstract

**Background:**

The Dubousset Functional Test (DFT) offers a standardized, objective assessment to evaluate physical performance, trunk and lower extremity muscle strength, balance, and motor-cognitive integration. However, the psychometric properties of DFT in the aging Chinese population remain unexamined. Therefore, this study aimed to determine the psychometric properties of the DFT in older Chinese adults.

**Methods:**

A cross-sectional psychometric design was employed, using convenience sampling. The DFT involves four components: (1) The Steps Test; (2) The Up and Walking Test; (3) The Down and Sitting Test; (4) The Dual-Tasking Test. To examine test-retest reliability, a subset of 20 participants was randomly selected from the total sample using a random number table and retested in a one-day interval. The 30 s Sit to Stand Test (30s-STS) was selected as the reference test to examine concurrent validity. Healthy control young adult participants were recruited to examine discriminant validity. SPSS version 26.0 and R software (version 4.1.0) were used to perform the data analysis.

**Results:**

Fifty participants were included in this study. Regarding the test–retest reliability, the ICC (95% CI) were 0.914 (0.756, 0.967), 0.985 (0.963, 0.994), 0.970 (0.926, 0.988), 0.964 (0.910, 0.986) for the steps test, the up and walking test, the down and sitting test, and the dual-tasking test, respectively. Moderate negative correlations were observed between the 30s-STS performance and the three components of the DFT: steps test (*r* = -0.45, *P* < 0.001), up and walking test (*r* = -0.52, *P* < 0.001), and down and sitting test (*r* = -0.51, *P* < 0.001). Subgroup analysis by age revealed that significant correlations were exclusively observed in the oldest old-aged group between the three components of the DFT (i.e., the steps test, up-and-walking test, and dual-tasking test) and the 30s-STS. Statistically significant differences were observed between older adults and healthy young adults for all components of DFT (all *P* < 0.001).

**Conclusion:**

The Dubousset Functional Test demonstrated relatively good psychometric properties for geriatric functional assessment.

## Background

Population aging has emerged as a critical global demographic phenomenon, representing both a hallmark of societal development and a significant public health challenge [1]. As highlighted in the World Population Aging 2020 report [2], the global population aged ≥ 65 years reached approximately 700 million in 2020, and this figure is projected to reach almost 1.5 billion by 2050. China, as one of the world’s most populous countries, is undergoing rapid demographic aging [3]. By 2030, China’s elderly population aged ≥ 65 years will reach 258 million, accounting for around 18% of the total population [4]. One significant consequence of demographic aging is the rising prevalence and incidence of chronic and degenerative diseases among older population, which substantially compromises quality of life in later years [5, 6].

Physical performance progressively declines with advancing age, particularly among older population [7]. Age-related physiological changes frequently lead to impaired balance control, affecting approximately 75% of individuals aged ≥ 70 years [8]. A growing body of research demonstrates that balance disturbance is the primary symptom of aging in older people [9, 10]. Furthermore, individuals aged more than 75 years have higher susceptibility to balance disorders, including age-related impaired balance and postural stability, benign paroxysmal positional vertigo, vestibular neuritis, and Meniere’s disease [11]. These conditions significantly compromise older adults’ functional independence by restricting daily activities performance and increasing the risk of falls [12]. It is reported that 30%~40% of older adults experience at least 1 fall annually, of whom half become recurrent fallers [13]. The fall-associated economic burden should not be ignored, with approximately $50 billion annual expenditures spent on non-fatal falls yearly [14]. These findings underscore the critical need for validated physical function and balance assessment tools tailored for Chinese older population to mitigate these adverse outcomes.

The assessment of balance and functional performance among older adults plays a vital role in identifying mobility limitations and reducing fall-related risk factors. Several functional assessment tools are commonly employed to evaluate balance and functional mobility in older population, such as the Berg Balance Scale (BBS) [15], Timed Up and Go Test (TUG) [16], and Dynamic Gait Index [17]. However, such aforementioned assessment tools demonstrate insufficient sensitivity to subtle changes in balance and functional performance ability. The Dubousset Functional Test (DFT), developed by Dr. Jean Dubousset, offers a standardized, objective assessment to evaluate physical performance, trunk and lower-extremity muscle strength, balance, and motor-cognitive integration [18]. The DFT comprises four key components: (1) getting up from an armless chair followed by a 5-meter forward and backward walk; (2) climbing steps; (3) descending to a seated position on the floor from a standing position; and (4) a dual-tasking test in which the participant walks while simultaneously counting down from 50. This test distinguishes itself from other functional performance tests by providing objective results about the individual’s functional performance and balance level, and measuring the adequate coordination, balance, attention, and thinking skills of individuals during function with the dual-task test component [19]. As reported by Diebo et al. [18], the reference values of the DFT derived from asymptomatic individuals were 15 (95% CI:14–16), 6.3 (95% CI: 6.0–6.6.0.6), 6.0 (95% CI: 5.4–6.6) and 13 (95% CI: 12–14) for the up and walking, steps, down and sitting, and dual-tasking tests, respectively. In addition, the psychometric properties of the DFT have been validated across diverse Turkish population, including patients with chronic non-specific low back pain [20], post-stroke survivors [21], individuals with early-stage Parkinson’s disease [22], and those with thoracic hyper-kyphosis [23]. However, the psychometric properties of the DFT remain unexamined in Chinese aging population, representing a significant gap in the validation of geriatric assessment instruments. Therefore, this study aimed to determine the psychometric properties of the DFT in older Chinese adults.

## Methods

### Participants

This study used a cross-sectional psychometric design and recruited participants from the Fuzhou Guode Elderly Care and Wellness Center, Fujian Province, China, using convenience sampling. The inclusion criteria were as follows: (1) age ≥ 60 years; (2) ability to ambulate independently without assistive devices or physical assistance; (3) adequate vision (corrected visual acuity ≥0.6) and hearing (ability to comprehend normal-volume speech) based on assessments documented in their latest routine physical examination reports; (4) cognitively intact measured by Montreal Cognitive Assessment (MoCA); (5) absence of severe organ dysfunction; (6) no history of acute cardiovascular events (e.g., myocardial infarction, unstable angina, or stroke) within the past six months. Participants were excluded if they had been diagnosed with dementia, an active infectious disease, or severe dysfunction of major organs (including cardiac, hepatic, cerebral, or renal systems). The inclusion criteria for the healthy young control adults were as follows: (1) aged between 18 and 30 years; (2) absence of self-reported or clinically diagnosed chronic diseases; (3) normal cognitive function, as indicated by a MoCA score within the normal range; (4) ability to independently complete all functional tests. Exclusion criteria included the presence of acute or chronic musculoskeletal conditions affecting mobility, a history of neurological or psychiatric disorders, or severe visual or hearing impairment that could not be corrected with aids. According to the sample size recommendations of Bujang and Baharum [24], a subset of 20 participants was randomly selected from the total sample using a random number table to examine test-retest reliability. These participants underwent a second evaluation (retest) conducted by the same trained evaluator in a one-day interval. Fifty-one healthy young adults (12 males and 39 females) were recruited as control participants from Fujian Medical University in Fujian Province, China. The control group had an age range of 20 to 22 years. This study was approved by the Fujian Medical University Biomedical Research Ethics Review Committee (2024 Fuyi Ethics Review No. 323). The data were collected in March 2025 and written informed consent was obtained from both older adults and healthy young adults before the study was formally conducted.

### Sample size computation

According to the recommendation for reliability analyses, 30–50 participants should be recruited in the study [25]. A priori power analysis was performed with the G*Power program (version 3.0.10; Universitat Dusseldorf, Dusseldorf, Germany) to determine the required minimum sample size before enrollment. In a similar study, ICC values were 0.819–0.965 [19]. Based on these values, the smallest sample size to be included in the study was 42, when the statistical significance of alpha was 5% and the power of the study (1-β) was 95%.

### Instruments

The Dubousset Functional Test (DFT) is a practical four-component global functional assessment conceptually proposed by Dr. Jean Dubousset [18]. The DFT involves four components: (1) The Steps Test: participants start 50 cm from the base of the stairs, ascend three consecutive stairs, turn around on the top stairs, and descend all three stairs; (2) The Up and Walking Test: participants stand up from a standard armless chair without assistance, walk 5 m forward before stopping, walk backwards the same distance, and return to sit down independently; (3) The Down and Sitting Test: participants lower themselves to a seated position on the ground from an upright position, then return to standing with assistance when necessary; and (4) The Dual-Tasking Test: participants walk 5 m forward, turn around, and walk 5 m back to the starting position while simultaneously performing a working memory task (serial subtraction of 2 from 50). The DFT was conducted by the research assistants who completed a minimum of 20 h of protocol training including theoretical instructions and demonstration sessions with standardized persons, followed by supervised practice until achieving more than 90% scoring accuracy against gold-standard ratings.

The 30 s Sit to Stand Test (30s-STS) was conducted using a standard 45-cm chair without armrests, stabilized by an evaluator to prevent sliding. Participants began seated with an upright posture, feet shoulder-width apart, and arms crossed over the chest [26]. Participants were instructed to perform maximal stand-sit repetitions within 30 s, achieving full knee and hip extension during standing while minimizing body weight support on the chair when sitting. If the participants were unable to maintain reduced weight support while sitting, they were permitted to perform the test conventionally with full weight discharge. The participants performed three consecutive trials with 1-minute rest intervals. Only complete sit-to-stand cycles were counted, and the mean score across trials was used for the analysis. The 30s-STS has been widely validated as a measure of functional strength, balance, and fall risk, supporting its use as a reference test in this context. It demonstrates significant associations with both static and dynamic balance in older adults [27, 28]. Moreover, the test is strongly correlated with several functional outcomes such as habitual gait speed (*r* = 0.517, *p* < 0.01), dynamic balance measured by Time Up and Go (TUG) test (*r* = − 0.501, *p* < 0.01), and endurance assessed via Six Minutes Walking Test (6MWT) (*r* = 0.558, *p* < 0.01) [29]. It also discriminates effectively between older adults with and without fall risk (AUC = 0.77) and correlates well with the Fullerton Advanced Balance (FAB) scale (*r* = 0.73, *p* < 0.001) [30]. Additionally, parameters such as duration, power and velocity derived from 30s-STS show strong correlations with Short Physical Performance Battery (SPPB) components and handgrip strength, especially in adults over 70 years old (*p* < 0.01) [31]. Although other assessment tools such as the SPPB and TUG provide valuable insights into specific functional domains, the 30s-STS was selected as the sole reference test because it integrates assessment of multiple functional domains, including strength, balance, mobility, and endurance, while remaining simple to administer. Its strong correlations with diverse functional outcomes support its use as a single efficient tool, reducing assessment complexity without compromising comprehensiveness.

### Statistical analysis

Data analysis was performed using SPSS version 26.0 (Chicago, IL, USA) and the R software (version 4.1.0; R Foundation for Statistical Computing, Vienna, Austria). Statistical significance was defined as a two-tailed *P*-value < 0.05. Missing data were analyzed using regression imputation. The Shapiro-Wilk test was used to examine the normality of the distribution of variables. Normally distributed continuous variables were presented as mean ± standard deviation ($\:\stackrel{-}{\text{X}}$±SD), whereas non-normally distributed data were reported as medians with interquartile ranges [Median (Q1, Q3)]. Categorical variables were described as frequencies (%).

Test-retest reliability was assessed using the intraclass correlation coefficient (ICC) with two-way random effects, absolute agreement, and a single-measurement model. The strength of the reliability was interpreted as follows: weak (ICC < 0.40), below moderate (ICC 0.40–0.59), moderate (ICC 0.60–0.74), good (ICC 0.75–0.89), and excellent (ICC ≥ 0.90) [32].

Concurrent validity was assessed using Spearman’s rank correlation coefficient (r). The DFT was evaluated against the 30s-STS. The strength of correlations was interpreted according to Dancey and Reidy’s classification criteria: 0.00 (no correlation), 0.001–0.29 (weak), 0.30–0.70 (moderate), 0.71–0.99 (strong), and 1.00 (perfect correlation) [33]. Discriminant validity was evaluated using the Mann Whitney U test to compare balance performance between older adults (decreased balance capacity) and young control adults (normal balance function).

## Results

A total of 50 participants including 17 males and 33 females with a mean age of 83.74 ± 5.23 were included in this study (detailed in Table 1). Most participants were non-partnered (58%) and presented with multimorbidity (76% with chronic conditions). 64% of participants reported poor vision, with 54% reporting poor hearing. Physical activity levels were generally low (76% engaged in < 30 min of daily exercise), although only 16% reported falls in the past year.

**Table 1:** Basic characteristics of included participants

Items	Older adults
Age (years),$\:\stackrel{-}{\text{X}}$±SD	83.74 ± 5.23
Sex, n (%)	
Male	33 (66.00)
Female	17 (34.00)
Educational level, n (%)	
Primary school or below	14 (28.00)
Middle school	7 (14.00)
High school or vocational school	22 (44.00)
Undergraduate or above	7 (14.00)
Marital status, n (%)	
Married or living with partner	21 (42.00)
Widowed, divorced, separated or never married	29 (58.00)
Past medical history, n (%)	
Yes (e.g., hypertension, diabetes, cardiovascular disease or arthritis)	38 (76.00)
No	12 (24.00)
Fall history in previous one year, n (%)	
Yes	8 (16.00)
No	42 (84.00)
Length of exercise, n (%)	
< 30 min	38 (76.00)
≥ 30 min	12 (24.00)
Vision condition, n (%)	
Good	18 (36.00)
Self-reported poor vision	32 (64.00)
Hearing condition, n (%)	
Good	23 (46.00)
Self-reported poor hearing	27 (54.00)

Regarding the test–retest reliability, the ICC (95% CI) were 0.914 (0.756, 0.967), 0.985 (0.963, 0.994), 0.970 (0.926, 0.988), 0.964 (0.910, 0.986) for the steps test, the up and walking test, the down and sitting test, and the dual-tasking test, respectively.

**Fig. 1:**
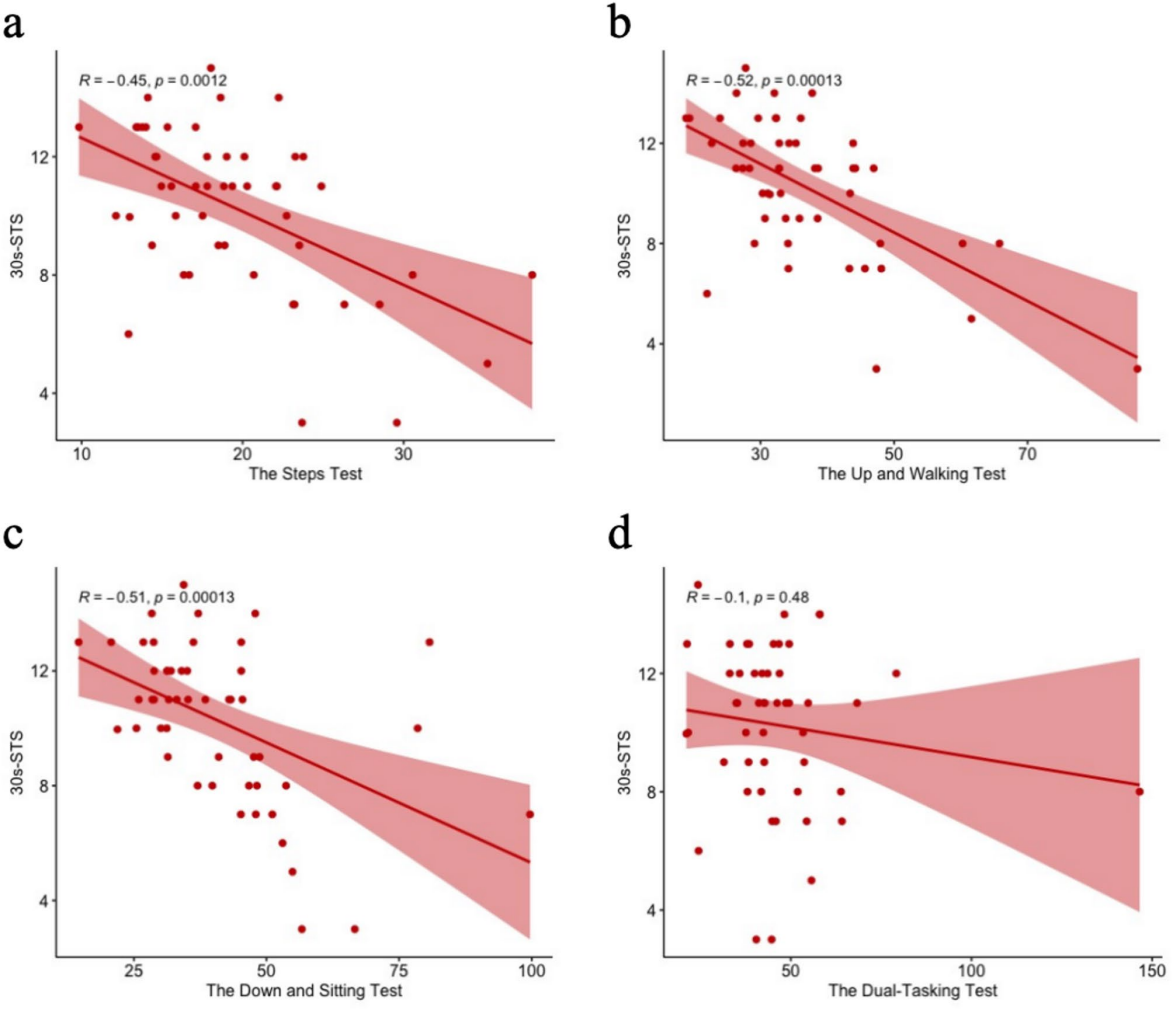
The correlations between the DFT and 30s-STS. a: the correlations between the steps test and 30s-STS; b: the correlations between the up and walking test and 30s-STS; c: the correlations between the down and sitting test and 30s-STS; d: the correlations between the dual-tasking test and 30s-STS

Figure 1 demonstrated the significant correlations between the DFT and 30s-STS. The analysis revealed moderate negative correlations between 30s-STS performance and the three components of the DFT: the steps test (*r* = −0.45, *P* < 0.001), the up and walking test (*r* = −0.52, *P* < 0.001), and the down and sitting test (*r* = −0.51, *P* < 0.001), indicating that participants with higher 30s-STS repetition counts demonstrated shorter completion times on these tests. However, no significant association was observed between the 30s-STS performance and dual-tasking test (*r* = −0.1, *P* = 0.48), suggesting that lower extremity strength, endurance, and balance, as measured by the 30s-STS, may not directly influence cognitive-motor integration performance.

Interestingly, when participants were subdivided into the middle old-aged group (70–80 years) and the oldest old-aged group (> 80 years), we found significant correlations between the three components of the DFT (i.e., the step test, the up and walking test, and the dual-tasking test) and the 30s-STS (as shown in Table 2). No significant associations were detected in the middle old-aged subgroup (all *P* > 0.05).

**Table 2:** Age-stratified subgroup analysis of correlations between four components of the DFT and 30s-STS

		30s-STS	
		70 ~ 80 years (*n* = 15)	> 80 years (*n* = 35)
The step test	r	−0.511	−0.446
	p	0.052	0.007
The up and walking test	r	−0.300	−0.548
	p	0.278	< 0.001
The down and sitting test	r	−0.389	−0.579
	p	0.152	< 0.001
The dual-tasking test	r	0.091	−0.127
	p	0.747	0.466

We compared the four components of the DFT between older adults and healthy young controls (Table 3). The analysis revealed statistically significant differences between groups for all components of the DFT (all *P* < 0.001). The effect sizes were large (*r* > 0.85). Older adults demonstrated markedly longer completion times (as shown by higher medians), and greater variability (as shown by wider interquartile ranges) compared to the young group.

**Table 3:** The differences of the DFT between older adults and healthy young adults

	Groups		*p*	Effect sizes (*r*)
	Older adults	Young adults		
The step test	18.56 (15.04, 23.04)	7.65 (7.23, 8.22)	< 0.001	0.861
The up and walking test	33.39 (29.24, 43.39)	12.54 (11.48, 13.49)	< 0.001	0.861
The down and sitting test	37.08 (31.21, 47.82)	4.80 (4.20, 5.32)	< 0.001	0.861
The dual-tasking test	43.20 (37.91, 51.32)	12.82 (11.07, 15.24)	< 0.001	0.857

Data were presented as median (25th percentile, 75th percentile)

## Discussion

This study aimed to examine the test-retest reliability, concurrent validity, and discriminant validity of the Dubousset Functional Test among older Chinese adults. These findings demonstrate that DFT is reliable and valid in older population. DFT demonstrates distinct practical advantages over conventional functional assessments, owing to its minimal equipment requirements (standard stopwatch and common household items) and brief administration time (< 5 min). This operational simplicity renders it particularly suitable for implementation in resource-constrained clinical environments and population-level screening programmes. Notably, this study makes a significant contribution to the literature by establishing the psychometric properties of DFT in older adults, particularly those aged over 80 years in China.

The results indicated that older adults were able to complete the DFT within five minutes, and the test required no specialized equipment. Given its time-efficient, low-cost, and easy-to-administer nature, DFT is a practical and efficient assessment tool that can be readily integrated into diverse clinical and community settings. The test-retest reliability of all four DFT components demonstrated excellent consistency, with ICC values of 0.914 for the steps test, 0.985 for the up and walking test, 0.970 for the down and sitting test, and 0.964 for the dual-tasking test. The test-retest reliability of DFT in older adults is consistent with that of other studies. For example, ICC values were between 0.899 and 0.984 (excellent agreement) in post-stroke patients [21], and ICC values were 0.91, 0.86, 0.89, and 0.89, respectively, for the subcomponents of the DFT for patients with chronic non-specific low back pain [20]. This pattern of findings suggested that the DFT maintained its measurement consistency across diverse populations with varying physical capabilities, reinforcing its robustness as a functional assessment tool.

Moderate negative correlations were observed between the three DFT components (i.e., the steps test, the up and walking test, and the down and sitting test) and 30s-STS performance (*r* = −0.45 to −0.52, all *p* < 0.001). The 30s-STS was employed as the reference criterion to establish the concurrent validity of the DFT in Chinese older adults. Although the 30s-STS is widely utilized in both clinical and research settings as a measure of lower limb strength [34], emerging evidence suggests that balance control significantly contributes to test performance [35–37]. The transitional movements between sitting and standing postures require precise dynamic balance control to maintain the body’s center of mass within the stability limits of the support base [27]. This principle of postural control operates bidirectionally: in the anteroposterior plane during rapid sit-stand transitions, and in the mediolateral plane to ensure symmetrical weight distribution between both lower extremities. Such dynamic stability is achieved through coordinated neuromuscular activation patterns during the 30s-STS performance. Biomechanical investigations utilizing force plate analysis have demonstrated that rapid sequential sit-to-stand performance serves as a sensitive indicator of fall history among older adults [38]. Importantly, these results reflect a decline in muscle power and a prolonged postural stabilization period, which effectively distinguished fallers from non-fallers. These findings were corroborated by Chorin et al., who demonstrated that older adults with a history of falls exhibited prolonged execution times during rapid sit-to-stand sequences and significantly reduced vertical and posterior-directed ground reaction forces compared to their non-faller counterparts [39]. Collectively, these findings supported that both lower-limb muscular power and dynamic postural control significantly contribute to the successful performance of rapid sit-to-stand movements.

Interestingly, no significant association was observed between performance on the 30s-STS and dual-tasking test (*r* = −0.1, *P* = 0.48). Dual tasking is a neurophysiological process that involves the simultaneous performance of two tasks, demanding integration between motor execution and cognitive function [40]. Campos-Magdaleno et al. found that performance on purely cognitive tasks (e.g., verbal fluency) under dual-task conditions was predominantly determined by cognitive domains, whereas performance on cognitive–motor tasks exhibited a broader dependence on psychosocial factors, general health status, and age [41]. The lack of a significant correlation between the 30s-STS and the DFT dual-tasking test may stem from the distinct cognitive-motor demands inherent to each task. The 30s-STS primarily assesses strength, balance, mobility, and endurance, with minimal cognitive load. In contrast, the DFT dual-tasking test explicitly challenges the integration of motor performance with concurrent cognitive input, requiring capacities such as attention, executive function, and motor control under divided attention [42]. This discrepancy suggests that the two tests capture largely independent constructs of functional ability. Taken together, the DFT offers distinct advantages over other tests for older population, as it captures integrated motor-cognitive performance across multiple functional domains.

Age-based subgroup analysis revealed a distinct pattern where three DFT components (i.e., the steps test, the up and walking test, and the dual-tasking test) correlated significantly with the 30s-STS only in the oldest-old (>80 years), but not in the middle-old (70–80 years). This suggests that the clinical and practical interpretation of the DFT may be age dependent. This finding of age-specific associations was consistent with earlier research of DFT in asymptomatic individuals. The study conducted by Diebo et al. reported that the time required to complete several DFT components, including the down and sitting (*r* = 0.529, *p* = 0.001), up and walking (*r* = 0.429, *p* = 0.001), and steps (*r* = 0.356, *p* = 0.014) tests, significantly increased with age [18]. This reinforces the notion that functional mobility, as evaluated by the DFT, declines with advancing age even in the absence of symptomatic disease. More broadly, the current results aligned with existing functional mobility literature. For instance, Butler et al. evaluated age-related differences across seven functional tests, such as stair ascent/descent and a 6-meter walk, and demonstrated that older adults performed significantly worse than younger participants across all measures [43]. The convergence of these findings across different cohorts and assessment tools underscores the pervasive impact of aging on functional capacity and supports the validity of the DFT as an age-sensitive measure.

The significant disparities between older adults and younger controls (all *p* < 0.001) provided strong evidence for the discriminant validity of the DFT. The substantially longer median completion times across all DFT components in older participants (e.g., the steps test: 18.56 vs. 7.65 s) provided quantitative evidence of characteristic age-related functional decline.

While this study provided valuable insights into the reliability and validity of DFT in older Chinese adults, several limitations should be considered. First, the study population was from a specific geographic region (China), which may limit the generalizability of the findings to other ethnic groups or institutionalized elderly population. Second, the interrater reliability could not be assessed due to the single-evaluator design of this study. Third, although the 30s-STS was used as a reference criterion for concurrent validity, comparisons with other established assessment tools (e.g., Short Physical Performance Battery, Timed Up & Go Test-Cognition) would have further strengthened the validity analysis. Fourth, the imbalance in sub-group sample size may reduce the statistical power for comparisons within the smaller subgroups. As such, the sub-group analysis should be interpreted with caution. Fifth, the substantial age gap between the older adult and young control groups may overstate the instrument’s actual capacity to distinguish more subtle, clinically relevant differences in function within older population or across narrower age ranges. As such, the discriminant validity of the DFT in this study should be interpreted with caution. Finally, the cross-sectional design of this study precludes the assessment of the measure’s responsiveness to change and its predictive validity for future outcomes.

## Conclusion

In conclusion, the Dubousset Functional Test demonstrated relatively good psychometric properties for geriatric functional assessments. Given its minimal equipment requirements and brief administration time (< 5 min), DFT is particularly suitable for implementation in resource-limited clinical and community screening settings.

## Data Availability

The datasets that support these findings are available from the corresponding author or the first author upon reasonable request.

## Abbreviations

DFT: Dubousset Functional Test
30s-STS: 30 s Sit to Stand Test
BBS: Berg Balance Scale
TUG: Timed Up and Go
SPPB: Short Physical Performance Battery
6MWT: Six Minutes Walking Test
FAB: Fullerton Advanced Balance
MoCA: Montreal Cognitive Assessment
ICC: Intraclass Correlation Coefficient

## References

[1] Khandelwal B, Gupta C. Leading causes of death and disability among the global aging community. In: Lama P, editor. The ageing population. Singapore: Springer; 2023.

[2] United Nations Department of Economic and Social Affairs, Population Division. World Population Ageing 2020 Highlights: Living arrangements of older persons. 2020. https://www.un.org/development/desa/pd/. Accessed 25 Aprial 2025.

[3] Fang EF, Xie C, Schenkel JA, Wu C, Long Q, Cui H, et al. A research agenda for ageing in China in the 21st century (2nd edition): focusing on basic and translational research, long-term care, policy and social networks. Ageing Res Rev. 2020;64:101174. 32971255 10.1016/j.arr.2020.101174 PMC7505078

[4] Xi JY, Liang BH, Zhang WJ, Yan B, Dong H, Chen YY, et al. Effects of population aging on quality of life and disease burden: a population-based study. Glob Health Res Policy. 2025;10(1):2. 39810282 10.1186/s41256-024-00393-8 PMC11731452

[5] Mitra S, Brucker DL. Disability and aging: from successful aging to wellbeing through the capability and human development lens. Disabil Health J. 2020;13(4):100924. 32354619 10.1016/j.dhjo.2020.100924

[6] Fong JH. Disability incidence and functional decline among older adults with major chronic diseases. BMC Geriatr. 2019;19(1):323. 31752701 10.1186/s12877-019-1348-z PMC6873710

[7] Alcazar J, Rodriguez-Lopez C, Delecluse C, Thomis M, Van Roie E. Ten-year longitudinal changes in muscle power, force, and velocity in young, middle-aged, and older adults. J Cachexia Sarcopenia Muscle. 2023;14(2):1019–32. 36788413 10.1002/jcsm.13184 PMC10067493

[8] Dillon CF, Gu Q, Hoffman HJ, Ko CW. Vision, hearing, balance, and sensory impairment in Americans aged 70 years and over: united States, 1999–2006. NCHS Data Brief. 2010;4(31):1–8. 20377973

[9] van der Veen SM, Thomas JS. A pilot study quantifying center of mass trajectory during dynamic balance tasks using an HTC vive tracker fixed to the pelvis. Sensors (Basel). 2021;21(23):8034. 34884036 10.3390/s21238034 PMC8659428

[10] Wang J, Li Y, Yang GY, Jin K. Age-related dysfunction in balance: a comprehensive review of causes, consequences, and interventions. Aging Dis. 2024;16(2):714–37. 38607735 10.14336/AD.2024.0124-1 PMC11964428

[11] Howcroft J, Kofman J, Lemaire ED. Review of fall risk assessment in geriatric populations using inertial sensors. J Neuroeng Rehabil. 2013;10(1):91. 23927446 10.1186/1743-0003-10-91 PMC3751184

[12] Matsumoto A, Yoshimura Y, Nagano F, Shimazu S, Shiraishi A, Kido Y, et al. Potentially inappropriate medications are negatively associated with functional recovery in patients with sarcopenia after stroke. Aging Clin Exp Res. 2022;34(11):2845–55. 36038811 10.1007/s40520-022-02224-7

[13] Ambrose AF, Paul G, Hausdorff JM. Risk factors for falls among older adults: a review of the literature. Maturitas. 2013;75(1):51–61. 23523272 10.1016/j.maturitas.2013.02.009

[14] Florence CS, Bergen G, Atherly A, Burns E, Stevens J, Drake C. Medical costs of fatal and nonfatal falls in older adults. J Am Geriatr Soc. 2018;66(4):693–8. 29512120 10.1111/jgs.15304 PMC6089380

[15] Simon A, Gyombolai Z, Kubik AZ, et al. Cross-cultural validation of the Berg balance scale to assess balance among Hungarian institutionalised older adults. Disabil Rehabil. 2024;46(13):2918–25. 38896556 10.1080/09638288.2023.2232717

[16] Brucki SM. Timed up and go test: a simple test gives important information in elderly. Arq Neuropsiquiatr. 2015;73(3):185–6. 25807121 10.1590/0004-282X20140243

[17] De Castro SM, Perracini MR, Ganança FF. Dynamic gait index–Brazilian version. Braz J Otorhinolaryngol. 2006;72(6):817–25. 17308836 10.1016/S1808-8694(15)31050-8 PMC9442121

[18] Diebo BG, Challier V, Shah NV, Kim D, Murray DP, Kelly JJ, et al. The dubousset functional test is a novel assessment of physical function and balance. Clin Orthop Relat Res. 2019;477(10):2307–15. 31135543 10.1097/CORR.0000000000000820 PMC6999954

[19] Abit KA, Sertel M, Aydoğan AS. Validity, reliability, and responsiveness of the Dubousset functional test in older adults. Top Geriatric Rehabilitation. 2023;39(3):197–202.

[20] Unver T, Unver B, Kacmaz KS. The test-retest reliability and minimal clinically important difference of the Dubousset functional test and its correlation with Rolland Morris disability questionnaire in chronic non-specific low back pain. Eur Spine J. 2023;32(6):2086–92. 37119310 10.1007/s00586-023-07720-6

[21] Bozkurt YE, Abit Kocaman A, Kaşıkcı Çavdar M, Keskin ED. A new instrument to assess physical function in stroke patients: the Dubousset function test and its validity, reliability, responsiveness. Neurol Res. 2023;45(12):1127–35. 37733422 10.1080/01616412.2023.2257439

[22] Abit Kocaman A, Aydoğan Arslan S, Bozkurt YE, Coşkun E. The Dubousset functional test: a reliable and valid test in early stage Parkinson’s disease patients. Neurol Sci. 2024;45(7):3137–46. 38296881 10.1007/s10072-024-07359-1 PMC11176096

[23] Unver T, Unver B, Sevik Kacmaz K. The psychometric properties of the Dubousset functional test in patients with thoracic hyperkyphosis. Eur Spine J. 2025. 10.1007/s00586-025-08830-z. 40216599 10.1007/s00586-025-08830-z

[24] Bujang MA, Baharum N. A simplified guide to determination of sample size requirements for estimating the value of intraclass correlation coefficient: A review. Archives Orofac Sci. 2017;12(1):1–11.

[25] Lexell JE, Downham DY. How to assess the reliability of measurements in rehabilitation. Am J Phys Med Rehabil. 2005;84(9):719–23. 16141752 10.1097/01.phm.0000176452.17771.20

[26] Jones CJ, Rikli RE, Beam WC. A 30-s chair-stand test as a measure of lower body strength in community-residing older adults. Res Q Exerc Sport. 1999;70(2):113–9. 10380242 10.1080/02701367.1999.10608028

[27] Monteiro PHM, Valenciano PJ, Mendes PHS, Teixeira LA. Association of 30-s sit-to-stand power test outcome with body balance in physically active older adults. J Aging Phys Act. 2025;33(4):370–8. 39753121 10.1123/japa.2023-0373

[28] Glenn JM, Gray M, Binns A. Relationship of sit-to-stand lower-body power with functional fitness measures among older adults with and without sarcopenia. J Geriatr Phys Ther. 2017;40(1):42–50. 26428899 10.1519/JPT.0000000000000072

[29] Yee XS, Ng YS, Allen JC, Latib A, Tay EL, Abu Bakar HM, et al. Performance on sit-to-stand tests in relation to measures of functional fitness and sarcopenia diagnosis in community-dwelling older adults. Eur Rev Aging Phys Act. 2021;18(1):1. 33419399 10.1186/s11556-020-00255-5 PMC7791746

[30] Roongbenjawan N, Siriphorn A. Accuracy of modified 30-s chair-stand test for predicting falls in older adults. Ann Phys Rehabil Med. 2020;63(4):309–15. 31520784 10.1016/j.rehab.2019.08.003

[31] Mahato NK, Davis A. Relationship between sit-to-stand movements and physical function in healthy older adults: testing duration power and displacement velocities for a 30-second chair-rise test. J Bodyw Mov Ther. 2025;42:139–45. 40325659 10.1016/j.jbmt.2024.12.022

[32] Mukaka MM. Statistics corner: A guide to appropriate use of correlation coefficient in medical research. Malawi Med J. 2012;24(3):69–71. 23638278 PMC3576830

[33] Dancey CP, Reidy J. Statistics without maths for psychology. London: Pearson education; 2007.

[34] Mehmet H, Yang AWH, Robinson SR. What is the optimal chair stand test protocol for older adults? A systematic review. Disabil Rehabil. 2020;42(20):2828–35. 30907166 10.1080/09638288.2019.1575922

[35] Jeon W, Jensen JL, Griffin L. Muscle activity and balance control during sit-to-stand across symmetric and asymmetric initial foot positions in healthy adults. Gait Posture. 2019;71:138–44. 31063929 10.1016/j.gaitpost.2019.04.030

[36] Lindemann U, Muche R, Stuber M, Zijlstra W, Hauer K, Becker C. Coordination of strength exertion during the chair-rise movement in very old people. J Gerontol A Biol Sci Med Sci. 2007;62(6):636–40. 17595420 10.1093/gerona/62.6.636

[37] Lord SR, Murray SM, Chapman K, Munro B, Tiedemann A. Sit-to-stand performance depends on sensation, speed, balance, and psychological status in addition to strength in older people. J Gerontol Biol Sci Med Sci. 2002;57(8):M539–43. 10.1093/gerona/57.8.m539 12145369

[38] Cheng YY, Wei SH, Chen PY, Tsai MW, Cheng IC, Liu DH, et al. Can sit-to-stand lower limb muscle power predict fall status? Gait Posture. 2014;40(3):403–7. 24974126 10.1016/j.gaitpost.2014.05.064

[39] Chorin F, Cornu C, Beaune B, Frère J, Rahmani A. Sit to stand in elderly fallers vs non-fallers: new insights from force platform and electromyography data. Aging Clin Exp Res. 2016;28(5):871–9. 26563286 10.1007/s40520-015-0486-1

[40] Woollacott M, Shumway-Cook A. Attention and the control of posture and gait: a review of an emerging area of research. Gait Posture. 2002;16(1):1–14. 12127181 10.1016/s0966-6362(01)00156-4

[41] Campos-Magdaleno M, Pereiro A, Navarro-Pardo E, Juncos-Rabadán O, Facal D. Dual-task performance in old adults: cognitive, functional, psychosocial and socio-demographic variables. Aging Clin Exp Res. 2022;34(4):827–35. 34648173 10.1007/s40520-021-02002-x PMC9076699

[42] Leone C, Feys P, Moumdjian L, D’Amico E, Zappia M, Patti F. Cognitive-motor dual-task interference: a systematic review of neural correlates. Neurosci Biobehav Rev. 2017;75:348–60. 28104413 10.1016/j.neubiorev.2017.01.010

[43] Butler AA, Menant JC, Tiedemann AC, Lord SR. Age and gender differences in seven tests of functional mobility. J Neuroeng Rehabil. 2009;6:31. 19642991 10.1186/1743-0003-6-31 PMC2741473

